# Complete Genome Sequence of Staphylococcus aureus EDCC 5398, a Clinical Isolate from Implant-Associated Bone Infection

**DOI:** 10.1128/MRA.01136-20

**Published:** 2020-10-22

**Authors:** Gopala Krishna Mannala, Markus Rupp, Hiren Ghosh, Torsten Hain, Eugen Domann, Volker Alt

**Affiliations:** a Department of Trauma Surgery, University Medical Centre Regensburg, Regensburg, Germany; b Institute for Medical Microbiology, Biomedizinsches Forschungzentrum Selterberg, Giessen, Germany; c German Center for Infection Research, Giessen-Marburg-Langen Partner Site, Giessen, Germany; University of Rochester School of Medicine and Dentistry

## Abstract

Here, we report the high-quality complete genome sequence of Staphylococcus aureus EDCC 5398, which was isolated from a patient with implant-associated bone infection. The assembled genome consists of 2,843,534 bp, with a G+C content of 33%; it has 2,739 coding sequences, belongs to sequence type 22, and contains *mecA* and *balz* genes, which contribute to methicillin resistance.

## ANNOUNCEMENT

Staphylococcus aureus is considered a major nosocomial opportunistic pathogen and is involved in skin and soft tissue infections to severe diseases such as endocarditis, toxic shock syndrome, and osteomyelitis. Approximately 30% of the human population carries S. aureus asymptomatically in anterior nares. However, this bacterium is also an opportunistic pathogen when the host defense system is breached. The clinical overuse of antibiotics to treat S. aureus infections had led to increases in antimicrobial-resistant strains to concerning levels (1).

S. aureus EDCC 5398 was isolated from an implant-associated bone infection and harbors methicillin resistance genes (2). S. aureus EDCC 5398 has been used as a methicillin-resistant Staphylococcus aureus (MRSA) model pathogen for studying various aspects of implant-associated infections (2). We determined to derive the complete genome sequence of S. aureus EDCC 5398 for use in comparative genomic and transcriptomic analyses with hybrid sequencing approaches, using short-read Illumina (San Diego, CA, USA) and long-read Pacific Biosciences (PacBio; Menlo Park, CA, USA) sequencing platforms.

The identification of S. aureus EDCC 5398 was established by API biochemical characteristic testing (bioMérieux, Marcy-l'Etoile, France) and 16S rRNA gene sequencing, and the strain was stored in 20% glycerol at −80°C (2). S. aureus EDCC 5398 was streaked onto LB agar from glycerol stock and grown overnight at 37°C. Later, a single colony was inoculated into 20 ml of LB medium and incubated overnight at 37°C with shaking at 180 rpm. The whole genomic DNA was isolated with the PureLink DNeasy kit (Invitrogen, Darmstadt, Germany) from an S. aureus EDCC 5398 culture grown overnight (<4 passages) and was used for sequencing on Illumina and PacBio platforms.

For Illumina sequencing, a library was constructed using the Nextera XT sample preparation kit, paired-end sequencing (2 × 300 bp) was performed on a MiSeq platform, and a total of 1,647,438 paired-end reads with a mean read length of 230 bp were obtained. Illumina read quality was initially checked using FastQC (scores of >Q30), and adapter sequences were trimmed using Trimmomatic. In this study, all software was used with default parameters unless otherwise specified. For long-read sequencing, a SMRTbell template library was prepared according to the instructions provided by PacBio. Whole-genomic DNA was sheared with g-TUBEs (Covaris, Brighton, UK) according to the manufacturer’s protocol. Size selection of the fragments was performed using 0.375× AMPure beads. For 10-kb library preparation, 10 μg of genomic DNA was end repaired and hairpin adapters were ligated with the DNA polymerase kit P4 (PacBio). The conditions for primer annealing and polymerase binding to the SMRTbell templates were determined by RS Remote, and sequencing was performed on the PacBio RS II platform. Quality control of the PacBio reads was performed with a minimum subread length of 500 bp and a minimum polymerase read quality of 0.8 using single-molecule real-time (SMRT) Portal v2.0.1. PacBio sequencing generated 150,292 prefiltered reads with an average read length of 1,251 bp.

*De novo* genome assembly was carried out with 34,906 filtered PacBio reads, with an average read length of 4,291 bp and an *N*_50_ value of 6,900 bp under the RS_HGAP_Assembly.3 protocol included in SMRT Portal v2.3.0. For error correction of the curated genome, Illumina short reads were mapped onto the final assembled genome using the Burrows-Wheeler Aligner (BWA) v0.6.2 (3), and subsequent variant and consensus sequence calling was performed using VarScan v2.3.6 (4) to obtain a highly accurate genome with a quality value (QV) of 60. The ends of the chromosomal contig were trimmed to remove artificial redundancies, and the chromosome was circularized with the *dnaA* gene in the initial position using BLAST. The annotation was initially performed by using Prokka v1.11 (5) and was checked with the NCBI Prokaryotic Genome Annotation Pipeline (PGAP) (6).

The complete closed chromosome of S. aureus EDCC 5398 consists of 2,843,534 bp with a G+C content of 33%, 2,739 coding sequences, 61 tRNAs, 19 rRNAs, and 2 intact prophage regions identified using PHAST (7).

*In silico* analysis using the multilocus sequence typing (MLST) tool (https://cge.cbs.dtu.dk/services/MLST) revealed that S. aureus EDCC 5398 belongs to sequence type 22 (ST22) (8). The genome of S. aureus EDCC 5398 includes multiple chromosomally encoded virulence genes (*sak*, *scn*, *hlgA*, *hlgB*, *hlgC*, and *aur*), enterotoxin genes (*seg*, *sei*, *sem*, *sen*, *seo*, and *seu*), and antimicrobial resistance genes (*norA*, *mecA*, and *balZ*) (9, 10). antiSMASH analysis suggests that EDCC 5398 contains nonribosomal peptide synthetase (NRPS) aureusimine and staphyloferrin (siderophore) biosynthetic gene clusters (11). We also found chromosomal mutations in the genes *gyrA* and *grlA*, which are known for ciprofloxacin resistance (12). The high-quality closed genome of S. aureus EDCC 5398 will be noteworthy for comparative genomic analyses, considering the genetic variability and the emergence of antimicrobial resistance and highly virulent strains in clinical settings.

### Data availability.

The complete genome sequence of S. aureus EDCC 5398 has been deposited in GenBank under accession number CP053101 under the BioProject number PRJNA630284. Raw reads from PacBio and Illumina sequencing have been submitted to the Sequence Read Archive (SRA) and are available under the accession numbers SRR12147540 and SRR12147095, respectively.
